# Development of an index that decreases birth weight, promotes postnatal growth and yet minimizes selection intensity in beef cattle

**DOI:** 10.5713/ab.23.0343

**Published:** 2024-01-20

**Authors:** Kenji Togashi, Toshio Watanabe, Atsushi Ogino, Masakazu Shinomiya, Masashi Kinukawa, Kazuhito Kurogi, Shohei Toda

**Affiliations:** 1 Livestock Improvement Association of Japan, Maebashi, Gunma 371-0121, Japan (Retired); 2 Livestock Improvement Association of Japan, Maebashi, Gunma 371-0121, Japan; 3 Livestock Improvement Association of Japan, Koto-ku, Tokyo 135-0041, Japan

**Keywords:** Birth Weight, Growth Curve, Index, Random Regression, Selection Intensity

## Abstract

**Objective:**

The main goal of our current study was to improve the growth curve of meat animals by decreasing the birth weight while achieving a finishing weight that is the same as that before selection but at younger age.

**Methods:**

Random regression model was developed to derive various selection indices to achieve desired gains in body weight at target time points throughout the fattening process. We considered absolute and proportional gains at specific ages (in weeks) and for various stages (i.e., early, middle, late) during the fattening process.

**Results:**

The point gain index was particularly easy to use because breeders can assign a specific age (in weeks) as a time point and model either the actual weight gain desired or a scaled percentage gain in body weight.

**Conclusion:**

The point gain index we developed can achieve the desired weight gain at any given postnatal week of the growing process and is an easy-to-use and practical option for improving the growth curve.

## INTRODUCTION

Genetic antagonism exists between the goal of rapid, efficient, early growth of food-animal progeny and the desire for small, low-maintenance parental strains [1–3]. In particular, it is important to reduce dystocia by decreasing the birth weight of progeny relative to the dam’s size. Expected responses to selection for body weight in cattle are a slightly greater increase in weight and degree of maturity at age of selection but also substantial increases in weight at all other ages, including maturity, due to positive genetic correlations between body weights throughout growth [4]. Therefore, restricted indices for selection on birth weight have been proposed for terminal sire lines used for heifers [5].

Achieving rapid growth to reach a specified final weight while reducing or maintaining birth weight is a significant challenge, not only from the perspective of the beef production industry, but also in terms of methane emissions to reduce climate change. A restricted selection index on birth weight cannot predict selection responses regarding the genetic gains for the entire fattening process unless the genetic correlations between birth and all other time points along the growth continuum are known. To address this issue, random regression (RR) models have been applied for the genetic evaluation of longitudinal data such as growth, lactation, and egg production curves [6–10]. In particular, RR models have been applied to analyze the entire fattening process [11–14], and a stage gain index based on RR coefficients, i.e., Legendre polynomials was developed for the lactation curve that minimized selection intensity [9].

In dairy cattle, the trait of interest is the stage increase in milk production, such as 305-day milk yield. However, most of the traits followed in beef cattle are genetic gains at specific time points such as weights at calving, as yearlings, and at slaughter. Achieving genetic weight gain at a specific point in the fattening process requires developing a point gain index. However, even though desired genetic gains at targeted times might be realized, multiple growth curves could achieve these results, especially when the time points of interest are far apart. Therefore, it will be important to obtain desired gains at various target times during the fattening process by applying minimum selection intensity and to develop optimal indices that predict the effects of achieving these time-point–specific gains throughout the entire fattening process.

Even when the same magnitude of genetic gain is achieved at a particular time point, the lower the intensity of selection, the lower the inbreeding coefficients [15]. In addition, the lower the selection intensity, the more likely the selection goal will be achieved. Using a low selection intensity provides the opportunity to increase the constraint by adding other time points to incorporate desired gains at target points during the fattening process. For example, an eigenvector index was developed to modify the lactation curve by using eigenvectors of genetic (co)variances of Legendre polynomial coefficients [16]. Therefore, here we compared point gain, eigenvector, and stage gain indices for their ability to achieve genetic gains at specific times during growth. An optimal index to fulfill desired gains at various time points during the fattening process can be constructed by using the genomically enhanced breeding value (GEBV) for Legendre polynomial coefficients [17–19]. The goal of our current study was to develop a selection index that achieved desired time-point–specific gains at the lowest selection intensity and that predicted the selection responses on weight gains throughout the entire fattening process. We provide an example to demonstrate the approach.

## MATERIALS AND METHODS

We used a RR model based on Legendre polynomials to develop our indices.

### Point gain index to achieve the desired gains at the specific times in fattening process with minimum selection intensity

The point gain index (*I_p_*) is described as


$$I_{p}=\sum_{j=0}^{k}b_{j}\;{GEBV}_{j}$$

where *b_j_* is the index weight for the jth order of Legendre polynomial coefficient, *GEBV_j_* is GEBV of the jth order of Legendre polynomial coefficient, and k is the order of Legendre polynomial function. In matrix notation, *I_p_* = ***b′GEBV****_αL_*, where ***b*** is a (k+1) vector of index weights and ***GEBV****_αL_* is a (k+1) column vector containing *GEBVj*(*j* = 0,1,..,k) for RR coefficients (***αL***). Hereafter *I_p_* refers to the point gain index.

Desired genetic gains (Δ***G****_s_*) at s specific times in the fattening process are described as


(1) $$S=\left[\begin{array}{ccccc} \phi_{0}(t_{1}) & \phi_{1}(t_{1}) & \phi_{2}(t_{1}) & .. & \phi_{k}(t_{1})\\ \phi_{0}(t_{2}) & \phi_{1}(t_{2}) & \phi_{2}(t_{2}) & .. & \phi_{k}(t_{2})\\ .. & .. & .. & .. & ..\\ \phi_{0}(t_{s}) & \phi_{1}(t_{s}) & \phi_{2}(t_{s}) & .. & \phi_{k}(t_{s}) \end{array}\right],\;\;\;S\Delta \alpha_{L}=\Delta G_{s},$$

and $\Delta G_{s}=[\begin{array}{cccc} \Delta G_{t1} & \Delta G_{t2} & .. & \Delta G_{ts} \end{array}]$, where Δ*G_ti_* is the desired genetic gain for the ith specific time during growth, ***S*** is a *s*×(*k*+1) matrix, *s* is the total number of restrictions in the fattening process, *t_i_* is the age standardized for the ith specific time in fattening process for the desired gains (*i* = *1*,..,*s*), *ϕ_j_*(*t_i_*) is the jth order of Legendre polynomial (*j* = *0*,..,*k*) evaluated at age *t_i_* standardized, and Δ***α****_L_* = (Δ*α_L_*__0__, Δ*α_L_*__1__, .., Δ*α_L_*_*_k_*_)′, where Δ*α_L_*_*_i_*_ is the difference in ith Legendre polynomial coefficient (*α_L_*_*_i_*_) before and after selection, i.e., Δ*α_L_*_*_i_*_ = *α_L_*_*_i_*_ after selection and −*α_L_*_*_i_*_before selection.

The vector of difference in Legendre polynomial coefficients after and before selection (Δ***α****_L_*) can be described according to BLUP properties [20] as


$$\begin{array}{l} \Delta \alpha_{L}=cov(\alpha_{L},I_{p})\frac{\bar{\iota }}{\sigma_{I_{p}}}\\ =cov(\alpha_{L},{\mathrm{GEBV}}_{\alpha L})b\frac{\bar{\iota }}{\sigma_{I_{p}}}=V_{{\mathrm{GEBV}}_{\alpha L}}b\frac{\bar{\iota }}{\sigma_{I_{p}}}, \end{array}$$

where ***α****_L_* is a [(*k*+1)×1] vector of true genetic Legendre coefficients, *V_GEBV_*_*_αL_*_ is the (co)variance matrix of ***GEBV***_*_αL_*_, *ῑ* is the intensity of selection, and *σ_I_*_*_p_*_ is the standard deviation of the point gain index (*I_p_*). The selection intensity (*ῑ*) required to achieve Δ***α****_L_* can be obtained by setting *ῑ* = *σ_I_*_*_p_*_. Therefore,


$$b={\left(V_{{\mathrm{GEBV}}_{\alpha L}}\right)}^{-1}\Delta \alpha_{L}$$

and


$$\begin{array}{l} \sigma_{I_{p}}^{2}=b^{\prime }V_{{\mathrm{GEBV}}_{\alpha L}}b\\ =\Delta {\alpha_{L}}^{\prime }{\left(V_{{\mathrm{GEBV}}_{\alpha L}}\right)}^{-1}V_{{\mathrm{GEBV}}_{\alpha L}}{\left(V_{{\mathrm{GEBV}}_{\alpha L}}\right)}^{-1}\Delta \alpha_{L}\\ =\Delta {\alpha_{L}}^{\prime }{\left(V_{{\mathrm{GEBV}}_{\alpha L}}\right)}^{-1}\Delta \alpha_{L}. \end{array}$$

The covariance GEBV between traits i and j was derived by assuming that sufficient data were used to estimate marker effects [21,22], such that


$$\begin{array}{l} cov(\alpha_{L},{\mathrm{GEBV}}_{\alpha L})=V_{{\mathrm{GEBV}}_{\alpha L}}\\ =\left[\begin{array}{cccc} r_{{GEBV}_{\alpha L_{0}}}^{2}\sigma_{g_{\alpha L_{0}}}^{2} & r_{{GEBV}_{\alpha L_{0}}}^{2}r_{{GEBV}_{\alpha L_{1}}}^{2}\sigma_{g-\alpha L_{0,1}} & \cdots & r_{{GEBV}_{\alpha L_{0}}}^{2}r_{{GEBV}_{\alpha L_{k}}}^{2}\sigma_{g-\alpha L_{0,k}}\\ r_{{GEBV}_{\alpha L_{1}}}^{2}r_{{GEBV}_{\alpha L_{0}}}^{2}\sigma_{g-\alpha L_{1,0}} & r_{{GEBV}_{\alpha L_{1}}}^{2} & \cdots & r_{{GEBV}_{\alpha L_{1}}}^{2}r_{{GEBV}_{\alpha L_{k}}}^{2}\sigma_{g-\alpha L_{1,k}}\\ \vdots & \cdots & \ddots & \vdots \\ r_{{GEBV}_{\alpha L_{k}}}^{2}r_{{GEBV}_{\alpha L_{0}}}^{2}\sigma_{g-\alpha L_{k,0}} & r_{{GEBV}_{\alpha L_{k}}}^{2}r_{{GEBV}_{\alpha L_{1}}}^{2}\sigma_{g-\alpha L_{k,1}} & \cdots & r_{{GEBV}_{\alpha L_{k}}}^{2} \end{array}\right], \end{array}$$

where $r_{GEBV\_\alpha L_{i}}^{2}$ is the reliability of the GEBV for the ith order of Legendre coefficient; $\sigma_{g\_\alpha L_{i}}^{2}$ is the genetic variance for the ith order of Legendre coefficient; and *σ_g_*_–_*_αL_*_*_i,j_*_ is the genetic covariance for the ith order and jth order of Legendre coefficients.

We used a Lagrange multiplier to choose a vector Δ***α****_L_* so that the index constructed based on Δ***G****_s_* has a minimum variance, with the restriction that the vector of expected genetic gains at specific *s* points during the fattening process ( $\Delta G_{s}^{*}$) is equal to the vector of desired genetic gains ( $\Delta G_{s}^{*}=(\begin{array}{cccc} \Delta G_{t1} & \Delta G_{t2} & .. & \Delta G_{ts} \end{array})$).

The function to be minimized is


$$f=\Delta {\alpha_{L}}^{\prime }{\left(V_{{\mathrm{GEBV}}_{\alpha L}}\right)}^{-1}\Delta \alpha_{L}+\lambda^{\prime }[S\Delta \alpha_{L}-\Delta G_{s}],$$

where ***λ*** = [***λ***_1_ ***λ***_2_ ….. ***λ****_s_*] is a vector of Lagrange multipliers.

Setting the partial derivatives of *f* with respect to Δ***α****_L_* equal to zero leads to


(2) $$\frac{\delta f}{\delta \Delta \alpha_{L}}=2{\left(V_{{\mathrm{GEBV}}_{\alpha L}}\right)}^{-1}\Delta \alpha_{L}+S^{\prime }\;\lambda =0.$$

Setting the partial derivatives of *f* with respect to ***λ*** equal to zero leads to


(3) $$\frac{\sigma f}{\sigma \lambda }=S\Delta \alpha_{L}-\Delta G_{s}=0.$$

Equations (2) and (3) can be written jointly as


(4) $$\left[\begin{array}{cc} 2{\left(V_{\mathrm{GEBV}\_\alpha L}\right)}^{-1} & S^{\prime }\\ S & 0 \end{array}\right]\;\left[\begin{array}{c} \Delta \alpha_{L}\\ \lambda \end{array}\right]=\left[\begin{array}{c} 0\\ \Delta G_{s} \end{array}\right].$$

According to the principle of Lagrange multipliers, the solution vector Δ***α****_L_* in equation (4) would lead to the minimum selection intensity and satisfy the constraints of the expected genetic gains being equal to the desired genetic gains.

The inverse of the coefficient matrix of equation (4) can be obtained through inversion by partitioning [23]. Therefore, the solution to equation (4) is


$$\left[\begin{array}{c} \Delta \alpha_{L}\\ \lambda \end{array}\right]=\left[\begin{array}{cc} \frac{1}{2}[V_{{\mathrm{GEBV}}_{\alpha L}}]-V_{{\mathrm{GEBV}}_{\alpha L}}S^{\prime }{\left({\mathrm{SV}}_{{\mathrm{GEBV}}_{\alpha L}}S^{\prime }\right)}^{-1}{\mathrm{SV}}_{{\mathrm{GEBV}}_{\alpha L}} & V_{{\mathrm{GEBV}}_{\alpha L}}S^{\prime }{\left({\mathrm{SV}}_{{\mathrm{GEBV}}_{\alpha L}}S^{\prime }\right)}^{-1}\\ {\left({\mathrm{SV}}_{{\mathrm{GEBV}}_{\alpha L}}S^{\prime }\right)}^{-1}{\mathrm{SV}}_{{\mathrm{GEBV}}_{\alpha L}} & -2{\left({\mathrm{SV}}_{{\mathrm{GEBV}}_{\alpha L}}S^{\prime }\right)}^{-1} \end{array}\right]\;\left[\begin{array}{c} 0\\ \Delta G_{s} \end{array}\right].$$

The first set of equations is equal to


(5) $$\Delta \alpha_{L}=V_{{\mathrm{GEBV}}_{\alpha L}}S^{\prime }{\left({\mathrm{SV}}_{{\mathrm{GEBV}}_{\alpha L}}S^{\prime }\right)}^{-1}\Delta G_{s}.$$

Conversely, Δ***α****_L_* in (5) has to satisfy the pre-specified gains as shown in (1), i.e., ***S***Δ***α****_L_* = Δ***G****_s_*. It can be proved as


$$S\Delta \alpha_{L}={\mathrm{SV}}_{{\mathrm{GEBV}}_{\alpha L}}S^{\prime }{\left({\mathrm{SV}}_{{\mathrm{GEBV}}_{\alpha L}}S^{\prime }\right)}^{-1}\Delta G_{s}=\Delta G_{s}.$$

Index coefficients (***b***) for the selection index based on Legendre polynomial coefficients are shown as


$$b={\left(V_{{\mathrm{GEBV}}_{\alpha L}}\right)}^{-1}\Delta \alpha_{L}.$$

Finally, the point gain index based on Legendre polynomial coefficients that would achieve the pre-specified gains with the least selection intensity is


$$I_{p}=\mathrm{GEB}V_{\alpha L}^{\prime }b=\mathrm{GEB}V_{\alpha L}^{\prime }[S^{\prime }{\left({\mathrm{SV}}_{{\mathrm{GEBV}}_{\alpha L}}S^{\prime }\right)}^{-1}\Delta G_{S}].$$

This is a general case when the number (s) of some specific points for desired gains is less than or equal to that of fitted Legendre coefficients (*k*+1), i.e., s≤*k*+1.

In particular, when *k*+1 = *s*, *S* is a square matrix, such that


$$\begin{array}{l} \Delta \alpha_{L}=V_{{\mathrm{GEBV}}_{\alpha L}}S^{\prime }{\left({\mathrm{SV}}_{{\mathrm{GEBV}}_{\alpha L}}S^{\prime }\right)}^{-1}\Delta G_{s}\\ =V_{{\mathrm{GEBV}}_{\alpha L}}S^{\prime }S^{\prime -1}{V_{{\mathrm{GEBV}}_{\alpha L}}}^{-1}S^{-1}\Delta G_{s}=S^{-1}\Delta G_{s}, \end{array}$$

then


$$S\Delta \alpha_{L}=\Delta G_{s}.$$

Therefore, Δ***α****_L_* in (5) achieves the desired gains at minimum selection intensity.

Note that many possible growth curves could satisfy the desired weight gains at the targeted times in the fattening process. The various indices derived from different sets of Δ***α****_L_* that satisfy the same vector of desired gains would have different selection intensities. This indicates that given a set of desired genetic gains, the solution to achieve the fixed set of genetic gains is not unique.

### Eigenvector index to achieve desired gains at specific time points in the fattening process by using the minimum selection intensity

The eigenvector index (*I_e_*) using n eigenvectors of the additive genetic RR covariance matrix is described as


$$I_{e}=\sum_{i=1}^{n}b_{i}\;(\sum_{j=0}^{k}e_{ij}\;{GEBV}_{j})=\sum_{i=1}^{n}b_{i}\;(\sum_{j=0}^{k}E_{ij}).$$

In matrix notation,


$$I_{e}={b^{\prime }}_{1\times n}\left[\begin{array}{c} {{ɛ}_{1}}^{\prime }\\ {{ɛ}_{2}}^{\prime }\\ ..\\ {{ɛ}_{n}}^{\prime } \end{array}\right]\;{\mathrm{GEBV}}_{\alpha L}={b^{\prime }}_{1\times n}E_{n\times 1},$$

where $E_{n\times 1}=\left[\begin{array}{c} {{ɛ}_{1}}^{\prime }{\mathrm{GEBV}}_{\alpha L}\\ {{ɛ}_{2}}^{\prime }{\mathrm{GEBV}}_{\alpha L}\\ ..\\ {{ɛ}_{n}}^{\prime }{\mathrm{GEBV}}_{\alpha L} \end{array}\right]$, ***ɛ****_i_* is a ith eigenvector of the additive genetic RR covariance matrix, i.e., covariance matrix of the additive genetic Legendre polynomial coefficients (*i* = 1,2,.., n), and the elements of ***ɛ****_i_* are shown as ${{ɛ}_{i}}^{\prime }=[\begin{array}{ccccc} e_{i0} & e_{i1} & e_{i2} & \ldots & e_{ik} \end{array}]$, where *e_ij_* is the jth element of ***ɛ****_i_*.

The variance of the eigenvector index is shown as


$$\sigma_{I_{e}}^{2}=b^{\prime }V_{E}b,$$

where ***V****_E_* is a (n×n) matrix and is shown as


$$V_{E}=\left[\begin{array}{cccc} {{ɛ}^{\prime }}_{1}V_{{\mathrm{GEBV}}_{\alpha L}}{ɛ}_{1} & {{ɛ}^{\prime }}_{1}V_{{\mathrm{GEBV}}_{\alpha L}}{ɛ}_{2} & .. & {{ɛ}^{\prime }}_{1}V_{{\mathrm{GEBV}}_{\alpha L}}{ɛ}_{n}\\ {{ɛ}^{\prime }}_{2}V_{{\mathrm{GEBV}}_{\alpha L}}{ɛ}_{1} & {{ɛ}^{\prime }}_{2}V_{{\mathrm{GEBV}}_{\alpha L}}{ɛ}_{2} & .. & {{ɛ}^{\prime }}_{2}V_{{\mathrm{GEBV}}_{\alpha L}}{ɛ}_{n}\\ .. & .. & .. & ..\\ {{ɛ}^{\prime }}_{n}V_{{\mathrm{GEBV}}_{\alpha L}}{ɛ}_{1} & {{ɛ}^{\prime }}_{n}V_{{\mathrm{GEBV}}_{\alpha L}}{ɛ}_{2} & .. & {{ɛ}^{\prime }}_{n}V_{{\mathrm{GEBV}}_{\alpha L}}{ɛ}_{n} \end{array}\right].$$

The genetic gains at specific times (Δ***G****_s_*) during the fattening process are described as


$$\Delta G_{s}=\left[\begin{array}{c} \Delta G_{t1}\\ \Delta G_{t2}\\ \Delta G_{t3}\\ .\\ \Delta G_{ts} \end{array}\right]=cov(G_{s},I_{e})\frac{\bar{\iota }}{\sigma_{I_{e}}},$$

where ***G****_s_* is a (*s*×1) vector of the true genetic values at s specific times during the fattening process, Δ*G_ti_* is the genetic gain at the ith time point in the fattening process, and *σ_I_*_*_e_*_ is the standard deviation of the eigenvector index (*I_e_*). In addition, *cov*(***G****_s_*,*I_e_*) can be described according to BLUP properties [20] as


$$\begin{array}{r} cov(G_{s},I_{e})=Scov(\alpha_{L},{\mathrm{GEBV}}_{\alpha L})[\begin{array}{cccc} {ɛ}_{1} & {ɛ}_{2} & .. & {ɛ}_{n} \end{array}]\left[\begin{array}{c} b_{1}\\ b_{2}\\ ..\\ b_{n} \end{array}\right]\\ =SV_{{\mathrm{GEBV}}_{\alpha L}}[\begin{array}{cccc} {ɛ}_{1} & {ɛ}_{2} & .. & {ɛ}_{n} \end{array}]\left[\begin{array}{c} b_{1}\\ b_{2}\\ ..\\ b_{n} \end{array}\right]=W_{s\times n}\left[\begin{array}{c} b_{1}\\ b_{2}\\ ..\\ b_{n} \end{array}\right]. \end{array}$$

The selection intensity (*ῑ*) required to achieve Δ***G****_s_* can be obtained by setting *ῑ* = *σ_I_*_*_e_*_. Therefore,


(6) $$W_{s\times n}\left[\begin{array}{c} b_{1}\\ b_{2}\\ ..\\ b_{n} \end{array}\right]=\Delta G_{s}.$$

We used a Lagrange multiplier (***λ***) to choose a vector ***b*** so that the index constructed based on Δ***G****_s_* has a minimum variance, with the restriction that the vector of expected genetic gains is equal to the vector of desired genetic gains. The function to be minimized is


$$f=b^{\prime }V_{E}b+\lambda^{\prime }[W_{s\times n}b-\Delta G_{s}].$$

Setting the partial derivatives of *f* with respect to ***b*** equal to zero leads to


$$\frac{\partial f}{\partial b}=2V_{E}b+{W^{\prime }}_{n\times s}\lambda =0.$$

Setting the partial derivatives of *f* with respect to ***λ*** equal to zero leads to


$$\frac{\partial f}{\partial \lambda }=W_{s\times n}b-\Delta G_{s}=0.$$

These equations can be written jointly as


$$\left[\begin{array}{cc} 2V_{E} & {W^{\prime }}_{n\times s}\\ W_{s\times n} & 0 \end{array}\right]\;\left[\begin{array}{c} b\\ \lambda \end{array}\right]=\left[\begin{array}{c} 0\\ \Delta G_{s} \end{array}\right].$$

Through inversion by partitioning [23], eigenvector index weights (***b***) are shown as


(7) $$b={V_{E}}^{-1}{W^{\prime }}_{\;n\times s}{\left(W_{s\times n}{V_{E}}^{-1}{W^{\prime }}_{n\times s}\right)}^{-1}\Delta G_{s}.$$

In addition, ***b*** in (7) has to satisfy the desired gains as shown in (6), i.e.,


$$W_{s\times n}b=\Delta G_{s}.$$

This can be proved as


$$W_{s\times n}b=W_{s\times n}{V_{E}}^{-1}{W^{\prime }}_{n\times s}{\left(W_{s\times n}{V_{E}}^{-1}{W^{\prime }}_{n\times s}\right)}^{-1}\Delta G_{s}=\Delta G_{s}.$$

### Stage gain index to achieve the desired gains at specific stages during the fattening process by using the minimum selection intensity

We developed the stage gain index (*I_s_*) based on RR coefficients to achieve the desired genetic gains at specific growth stages by using the lowest possible selection intensity. The stage gain index (*I_s_*) is described in the same way as the point gain index (*I_p_*), that is


$$I_{s}=b^{\prime }{\mathrm{GEBV}}_{\alpha L}.$$

In the previous section regarding the point gain index (*I_p_*), ***SΔα****_L_*= Δ***G****_s_*.

However, in the current section regarding the stage gain index (*I_s_*), the vector Δ***G****_s_* is the desired genetic gains for s stages, and


$$\Delta G_{s}=(\Delta G_{s1},\Delta G_{s2},\ldots ,\Delta G_{ss}),$$

where Δ*G_sj_* is the desired genetic gain for the jth stage in the fattening process. Note that Δ*G_sj_* is not the jth specific time point during growth but the jth specific stage during the fattening process. Therefore, ***S*** does not correspond to a specific point but to a specific stage during the fattening process. That is, ***S*** is described as


$$S=\left[\begin{array}{cccc} \sum_{t=m_{1}}^{n_{1}}\phi_{0}(t) & \sum_{t=m_{1}}^{n_{1}}\phi_{1}(t) & .. & \sum_{t=m_{1}}^{n_{1}}\phi_{k}(t)\\ \sum_{t=m_{2}}^{n_{2}}\phi_{0}(t) & \sum_{t=m_{2}}^{n_{2}}\phi_{1}(t) & .. & \sum_{t=m_{2}}^{n_{2}}\phi_{k}(t)\\ \cdots & \cdots & \cdots & \cdots \\ \sum_{t=m_{s}}^{n_{s}}\phi_{0}(t) & \sum_{t=m_{s}}^{n_{s}}\phi_{1}(t) & \cdots & \sum_{t=m_{s}}^{n_{s}}\phi_{k}(t) \end{array}\right],$$

where *ϕ_i_*(*t*) is the ith order of Legendre polynomial evaluated at week *t* standardized, and *m_j_* and *n_j_* are the first and last week of age of the jth stage, respectively. In this study, the fattening process was measured in units of weeks of age; accordingly stage was divided into units of weeks of age. Note that the only difference between the point gain and stage gain indices is the definition of *S*. The point gain index and stage gain index correspond to the genetic gains for specific points and specific stages, respectively; all other equations are completely the same between these two indices. Therefore, as in the previous section on the point gain index (*I_p_*), the difference in Legendre polynomial coefficients (Δ***α****_L_*) (*α* after selection – *α* before selection) in the stage gain index (*I_s_*) can be described as


$$\Delta \alpha_{L}=V_{{\mathrm{GEBV}}_{\alpha L}}S^{\prime }{\left({\mathrm{SV}}_{{\mathrm{GEBV}}_{\alpha L}}S^{\prime }\right)}^{-1}\Delta G_{s}.$$

Then the stage gain index (*I_s_*) is shown as


$$I_{s}=\mathrm{GEB}V_{\alpha L}^{\prime }b=\mathrm{GEB}V_{\alpha L}^{\prime }[S^{\prime }{\left({\mathrm{SV}}_{{\mathrm{GEBV}}_{\alpha L}}S^{\prime }\right)}^{-1}\Delta G_{s}].$$

As mentioned previously about the point gain index, we can choose an ideal unique stage gain index to achieve the desired gains at specific stages by using a minimum selection intensity when the number (s) of restrictions or desired gains is less than the number of Legendre polynomial coefficients (*k*+1), i.e., *s*≤*k*+1.

### Numerical example

We assumed the genetic covariance matrix of Legendre polynomial coefficients (Table 1) given the fattening process in Japanese Black steers [24,25] (Supplementary file). In the current study, quartic Legendre polynomials (*k* = 4) were assumed as done previously [26,27,13]. Japanese Black steers are slaughtered at approximately 30 months of age [28], so we fitted a growth curve to 130 weeks of age, corresponding to 30 months of age. We assumed that the growth curve before selection was similar to the curve from [24], who fitted a Gompertz growth curve. Instead, we fitted a RR model for that curve to develop a selection index based on RR coefficients. Gompertz growth curve depends on three parameters, i.e., A, B, and K are the asymptotic weight, growth starting point, and maturity rate of the growth curve, respectively. The three parameters, A, B, and K are used from [24] such as 768, 3.4, and 0.03, respectively. Weekly body weights from birth through 130 weeks of age were estimated from Gompertz growth curve by using the three parameters [24]. Covariates for Legendre RR curve are shown as ***ϕ*** in Supplementary file. RR coefficients before selection are estimated from weekly body weights from birth through 130 weeks of age and covariates for Legendre RR curve. The desired curve was derived according to the breeding goal that the birth weight was less than before selection and that the 130-week-old weight was achieved earlier than before selection. Birth weight; body weight at 5, 81, 127, 128, and 130 weeks of age during the fattening process; and the Legendre coefficients before selection and those of the desired curve are shown (Table 2).

The desired gain at a specific point or a stage can be any value that satisfies the breeder’s purpose. However, we assumed a desired curve as a criterion, to compare the point gain, stage gain, and eigenvector indices and to show that the procedures we developed in this study are correct. We set four break points at weeks of age during the fattening process from birth to 130 weeks of age and used four combinations of break points, such that combination 1 = 0, 40, 120, and 130 weeks; combination 2 = 0, 26, 106, and 130 weeks; combination 3 = 0, 26, 120, and 130 weeks; and combination 4 = 0, 42, 86, and 130 weeks. The specific weeks of age chosen for combination 4 roughly divide the entire 130-week growth period into thirds. The time points of 40 and 120 weeks were derived from the inflection points of the growth curve before selection (Table 2). The times of 26 and 106 weeks were derived from the inflection points of the first eigenvector function for the covariance matrix of the genetic Legendre coefficients (Table 1).

The desired gains at selected specific points and stages were computed from the difference between the body weight from the desired growth curve and that before selection (i.e., BW in desired growth curve – BW before selection) (Table 2). The desired growth curve was chosen such that the body weight at 130 weeks of age before selection could be achieved approximately 2 weeks earlier. Similarly, the desired growth curve was chosen such that body weight at birth was approximately 2.5 kg less than that before selection. The genetic gain during the targeted stage was computed from the difference between the desired curve and that before selection. For example, during the specific stage from birth through 41 weeks of age, the desired stage gain $=\sum_{\text{i}=0}^{\text{i}=41}\text{BWi}$ in the desired curve $-\sum_{\text{i}=0}^{\text{i}=41}\text{BWi}$ before selection.

Regarding the eigenvector index, we had five eigenvectors because the order of matrix of Legendre coefficients is *k*+1, i.e., 5. We could provide a maximum of 5 index traits as products between the eigenvector and Legendre coefficients expressed in GEBV, that is,


$$({{ɛ}_{1}}^{\prime }{\mathrm{GEBV}}_{\alpha L},{{ɛ}_{2}}^{\prime }\mathrm{GEBV}\alpha L,\ldots ,{{ɛ}_{5}}^{\prime }{\mathrm{GEBV}}_{\alpha L}).$$

We compared two, three, four, and five index traits in the eigenvector index, i.e.,


$$\begin{array}{l} \text{two traits}={{ɛ}_{1}}^{\prime }{\mathrm{GEBV}}_{\alpha L}\;\text{and }{{ɛ}_{2}}^{\prime }{\mathrm{GEBV}}_{\alpha L};\\ \text{three traits}={{ɛ}_{1}}^{\prime }{\mathrm{GEBV}}_{\alpha L},\;{{ɛ}_{2}}^{\prime }{\mathrm{GEBV}}_{\alpha L},\;\text{and }{{ɛ}_{3}}^{\prime }{\mathrm{GEBV}}_{\alpha L};\\ \text{four traits}={{ɛ}_{1}}^{\prime }{\mathrm{GEBV}}_{\alpha L},\;{{ɛ}_{2}}^{\prime }{\mathrm{GEBV}}_{\alpha L},\;{{ɛ}_{3}}^{\prime }{\mathrm{GEBV}}_{\alpha L},\;\text{and }{{ɛ}_{4}}^{\prime }{\mathrm{GEBV}}_{\alpha L};\;\text{and}\\ \text{five traits}={{ɛ}_{1}}^{\prime }{\mathrm{GEBV}}_{\alpha L},\;{{ɛ}_{2}}^{\prime }{\mathrm{GEBV}}_{\alpha L},\;{{ɛ}_{3}}^{\prime }{\mathrm{GEBV}}_{\alpha L},\;{{ɛ}_{4}}^{\prime }{\mathrm{GEBV}}_{\alpha L},\;\text{and }{{ɛ}_{5}}^{\prime }{\mathrm{GEBV}}_{\alpha L}. \end{array}$$

We examined the effects of point gain selection on body weights throughout the entire fattening process by comparing the point gain index with the stage gain index when the number of stages was 1, i.e., the target period was the entire process. The reliability of *GEBV_j_* (*j* = 0, 1,.., *k*) for the jth order of Legendre polynomial coefficients was assumed to be 0.7. From the point that inaccurate estimation of population parameters could bias estimates of theoretical gains, in addition to the reliability of 0.7, we also added reliability of 0.5 and 0.6 to the point gain selection index in which the four target ages were 0, 42, 86, and 130 weeks and the respective desired gains were −2.5, 15.7, 16.6, and 5.4 kg.

## RESULTS AND DISCUSSION

### Gains achieved by using the point gain, stage gain, and eigenvector indices

We set 4 combinations of specific weeks of age for the desired gains in the numerical example. Therefore, we had a 4-point gain index, with four specific time points (weeks of age) throughout the fattening process; these four time points divided the fattening process into three stages and thus created a 3-stage gain index. The target ages in the 4 combinations of 3-stage and 4-point gain indices are shown (Table 3). The restrictions on birth weight and body weight at 130 weeks of age were the same among the 4 combinations. The selection intensity and achieved body weight (Table 4), which is the sum of the weight before selection and genetic gain, are based on the point gain, eigenvector, and stage gain indices (Table 3). Each of the 4 combinations represents 3 stages from birth through 130 weeks of age. For example, the four ages selected for combination 1 (0, 40, 120, and 130 weeks) yields the 3 stages of 0 through 39 weeks, 40 throughout 119 weeks, and 120 through 130 weeks. The difference between the achieved genetic gain due to index selection at a specific point or stage and the desired genetic gain was zero (Table 4), indicating that the indices we developed were valid. Because the order of the covariance matrix of Legendre polynomial coefficients (*k*+1 = 5) is greater than the number of restrictions or desired gains at the specific points (4) or stages (3), the index with the lowest selection intensity is uniquely selected. Thus, the point gain and eigenvector indices each achieved a 2.5 kg lower birth weight. Moreover, these indices achieved the same body weight at 130 weeks of age as before selection (720.4 kg) but approximately 2 weeks earlier (Table 4).

The selection intensity and achieved body weights due to the point gain index were completely the same as those of the eigenvector index with five index traits, because all five eigenvectors were derived from the covariance matrix of Legendre polynomial coefficients. The selection intensity of the stage gain index was greater than that of the point gain index (Table 4), because restriction by the stage gain index would be stricter than that by the point gain index. That is, restriction by the stage gain index involves the long-term weight gains during the fattening process, whereas restriction by the point gain index addresses a specific time point along the fattening process. Weights at birth and at 130 weeks were lower for the stage index than the point or eigenvector gain indices. Therefore, whether the point gain index or stage gain index is preferable depends on whether breeders prefer to model genetic gains at a specific age or at a particular growth stage. As is the practice for beef fattening, if breeders want to increase body weight at a particular age, the point gain index would be preferable. However, if breeders want to model long-term weight gains such as those during the early, middle, and late stages of fattening, the stage gain index would be preferable.

The restrictions on the birth weight and body weight at 130 weeks were the same among the 4 combinations, such that the birth and finishing weights were identical among 4 combinations (Table 4). Even though the restriction on weight gains throughout the fattening process, except for those at birth (week 0) and during the final fattening week (week 130), differed among the 4 combinations (Table 3), the weight gains at 5, 81, 127, and 128 weeks were almost the same among all combinations (Table 4). The time points of 0, 42, 86, and 130 weeks (combination 4) essentially divided the entire fattening process into thirds. Dividing the fattening process into equal periods and assigning these dividing points to specific weeks of age would be a viable option, although the weeks chosen as targeted points for desired gains would be at the breeder’s discretion.

### Comparison of the number of index traits in eigenvector selection

The selection intensity, birth weight, and body weight at 5, 81, 127, 128, and 130 weeks of age by using the eigenvector and point gain indices are shown (Table 5). We had five eigenvectors because the order of the covariance matrix of Legendre polynomial coefficients was five. The first, second, and third eigenvectors explained 83.8%, 15.9%, and 0.3% of the genetic variation during the whole fattening process, whereas the fourth and fifth eigenvectors explained almost 0%. Therefore, as mentioned regarding the numerical example, we set four combinations of eigenvectors. The first and the second eigenvectors are treated as index traits when the eigenvector number is 2 (Table 5). That is, the two index traits are **ɛ_1_′GEBV_αL_** and **ɛ_2_′GEBV_αL_**. In the same way, the first, second, and third eigenvectors are treated as index traits when the eigenvector number is 3 (Table 5), that is, the three index traits are **ɛ_1_′GEBV_αL_**, **ɛ_2_′GEBV_αL_**, and **ɛ_3_′GEBV_αL_**, and so on for all four conditions.

The difference between the desired and achieved weight gains was zero, demonstrating that our developed method was valid. As mentioned earlier, the eigenvector index and point gain index yield identical results when all eigenvectors are derived from the covariance matrix of Legendre polynomial coefficients. Consequently, the selection intensity and all body weights throughout growth (Table 5) were the same between the eigenvector index with 5 index traits and the point gain index. The proportion of eigenvector selection that explains the genetic variance throughout the fattening process—represented as the genetic covariance matrix of Legendre polynomial coefficients—decreases as the number of index traits decreases, that is, from 5 to 4, from 4 to 3, and from 3 to 2.

The selection intensity increased as the number of index traits decreased, indicating that the rigor of selection had to increase to achieve the same genetic gains as the proportion of eigenvector selection that explains the genetic variance throughout the fattening process decreased. Furthermore, the deviation of body weights at 5 and 81 weeks of age from eigenvector selection using all five index traits increased as the number of index traits decreased. Therefore, eigenvector index selection when the number of index traits was less than the order of matrix of Legendre polynomial coefficients was inferior to both the eigenvector index derived by using the covariance matrix of Legendre coefficients and to the point gain index.

For the purpose of modeling increasing growth while reducing or maintaining birth weight, we set the target ages at 0 weeks and 130 weeks, with respective desired gains of −2.5 and 5.4 kg. We computed these desired gains according to the differences in body weight between the desired curve and that before selection (Table 2). All of the eigenvector indices achieved the same desired gain, such that the birth weight (24.9 kg) and weight at 130 weeks of age (725.8 kg) were the same among all indices.

**Figure 1:**
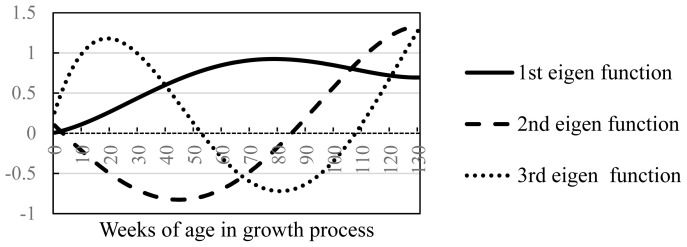
Eigen function in Legendre function.

The eigen functions for the first, second, and third eigenvectors are shown (Figure 1). The first eigen function increased until approximately 80 weeks of age and then declined gradually. The second eigen function was negative from birth until around 80 weeks of age and then increased from around 80 to 130 weeks of age (final fattening age). The eigenvector index likely helps to improve the growth curve by exploiting the properties of the first and second eigenvectors rather than the weight gains at particular ages during the fattening process.

### Selection intensity and Legendre coefficients at different target weight gains

The selection intensity, difference in Legendre coefficients before and after selection (Δ***α****_L_*), and index weights for stage, point gain, and eigenvector indices with different desired increases (listed as restriction values), are shown (Table 6). The number of stages in the stage gain index is one (Table 6), i.e., the entire fattening period is a single stage. The genetic gain for the entire fattening period was 1,490 kg, which we calculated from the Legendre function (Table 2), that is, ( $\sum_{\text{i}=0}^{\text{i}=130}$ ody weight of i week in the desired curve $-\sum_{\text{i}=0}^{\text{i}=130}$ body weight of i week before selection). In contrast, we arbitrarily set stage gain to 1,000 kg. The selection intensity, difference in Legendre coefficients before and after selection (Δ***α****_L_*), and index weights calculated based on the restriction value of 1,490 were 1.49 times greater than those calculated by using the restriction value of 1,000. In the point gain index in which the four target ages were 0, 42, 86, and 130 weeks, the respective desired gains were −2.5, 15.7, 16.6, and 5.4 kg. However, the desired gains changed to −1, 6.28, 6.64, and 2.16 kg when the desired increase in birth weight was represented by −1.

The eigenvector index having two eigenvectors as index traits and two target ages (0 and 130 weeks) yielded desired gains of −2.5 kg at 0 weeks and 5.4 kg at 130 weeks. However, these desired gains changed to −1 and 2.16 kg when the desired increase was adjusted (i.e., scaled) for the increase in birth weight represented by −1. For both the point gain and eigenvector indices, the selection intensity, difference in Legendre coefficients before and after selection (Δ***α****_L_*), and index weights calculated by using a birth weight restriction value of −2.5 were 2.5 times larger than those calculated based on a birth weight restriction value of −1. The relationship between these indices is that the directions of the Legendre coefficients (Δ***α****_L_*) and index weights are multiplied by a constant only. Therefore, the results (Table 6) show that genetic gain can be expressed as actual weight gain or as any scaled value and that these indices are essentially the same.

### Effects of point gain index selection on body weights throughout the fattening process

**Figure 2:**
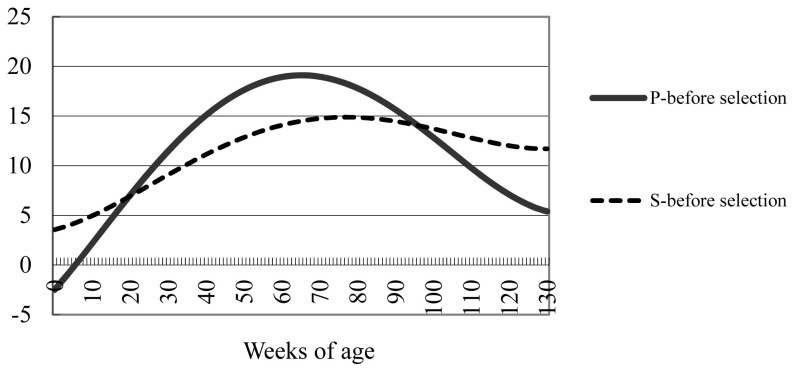
Deviation in body weight (kg) from that before selection for 1-stage gain index (S) and 4-point gain index (P).

As we mentioned in the numerical example, the effects of the point gain index selection on body weights throughout the fattening process are revealed by comparing the point gain index with the stage gain index that uses a single stage (i.e., the entire 130-week process) provided that the total weight gain during the fattening period is the same. The point gain index uses target ages of 0, 42, 86, and 130 weeks, with respective gains set to −2.5, 15.7, 16.6, and 5.4 kg (Table 3). The deviations of the stage gain and point gain indices from the growth curve before selection (Table 2) are shown (Figure 2). As we mentioned earlier regarding the stage gain index, the desired gain in body weight is the body weight throughout growth after selection less that before selection (Table 2). The stage gain index (Figure 2) has no restriction regarding desired gain at specific weeks of age. Therefore, the selection intensity was more moderate for the stage gain index (0.29) than the point gain index (0.40).

The effects of point gain index selection with restriction for desired gains at specific ages are clearly shown (Figure 2). In the point gain index, the restriction on body weight at birth was −2.5 kg, such that body weight was lower for the point gain index than for the stage gain index from birth until about 20 weeks of age. Based on the point gain index, the desired increases in body weight at 42 and 86 weeks of age were 15.7 and 16.6 kg, respectively, such that the point gain index yielded a greater body weight than the stage gain index from approximately 20 through 95 weeks of age. Thereafter the point gain index produced a smaller gain than the stage gain index: the magnitude of the desired gain at 130 weeks of age (i.e., final week of fattening) was 5.4 kg, which was much smaller than the desired gains at 42 weeks (15.7 kg) and 86 weeks (16.6 kg). In this way, the effects of point gain index selection on body weight throughout the entire fattening process can easily be grasped by comparing the point gain index with the stage gain index in which the weight gain over the entire fattening process (i.e., a single stage) was targeted.

In general, the more severe the restriction imposed, the greater the selection intensity required to realize the restriction. For practical reasons, the index with the lowest selection intensity should be chosen from among all possible indices that satisfy the same desired constraint, because the smaller the selection intensity, the greater the likelihood of realizing the selection goal. In this study, we showed that the point gain, stage gain, and eigenvector indices that we developed each provided only a single unique index that satisfies the pre-conditions and minimizes the selection intensity. An important point is which combination is optimal, i.e., which combination incorporates the lowest selection intensity when achieving the desired gains. Therefore, the necessary index to develop is one that minimizes selection intensity insofar as possible to achieve the desired gains at target time points during the fattening process and that, in doing so, yields a single, unique solution (*k*+1≥*s*). In contrast, when *k*+1<*s*, there are too many restrictions (*s*) regarding desired gains (Δ***G****_s_*) to satisfy the condition that ***S***Δ***α****_L_* = Δ***G****_s_*.

The procedure developed in this study allows comparing different genomic indices to select the most effective growth curve under given genetic parameters. On the other hand, the influence of errors of parameter estimation on the accuracy of the selection index has been investigated by Harris [29] and Heidhues [30]. The general conclusions by these re-searchers were that errors of parameter estimation would affect the expected response due to the selection index. Therefore, the effect of the difference in reliability of GEBV on selection response was investigated. Index coefficients, difference in Legendre coefficients (after selection – before selection, Δ***α****_L_*), and selection intensity in reliability of GEBV (0.7,0.6, and 0.5) are shown in Table 7. The absolute value of index coefficients increased as reliability of GEBV decreased from 0.7 to 0.5. Selection intensity increased with decreasing reliability of GEBV, since variance of index increases with an increase in index coefficients and variance is the square of selection intensity (*ῑ* = *σ_I_*_*_p_*_). The increase in GEBV prediction error associated with the decrease in reliability may have necessitated a slightly higher selection intensity to achieve the intended gain. However, difference in RR coefficients (after selection – before selection, Δ***α****_L_*) was almost the same despite the difference in reliability of GEBV. As a natural result, the amount of weight gain at each age from birth through 130 weeks was almost the same regardless of the difference in reliability of GEBV (not shown). Of course, the intended weight gain was achieved at all three reliability values of GEBV. In this study, Δ***α****_L_* was expressed as (5), i.e., Δ***α****_L_* = ***V****_GEBV_*_*_αL_*_***S*′**(***SV****_GEBV_*_*_αL_*_***S*′**)**^−1^**Δ***G****_s_*. The terms of ***V****_GEBV_*_*_αL_*_***S*′** and (***SV****_GEBV_*_*_αL_*_***S*′**)**^−1^** could have canceled out the effects on ***V****_GEBV_*_*_αL_*_, thereby reducing the effect of genetic parameter (***V****_GEBV_*_*_αL_*_) on Δ***α****_L_*. This study minimized index variance ( $\sigma_{I_{p}}^{2}=b^{\prime }V_{{\mathrm{GEBV}}_{\alpha L}}b$), as a result, the effect of genetic parameter (***V****_GEBV_*_*_αL_*_) onΔ***α****_L_* could have been decreased. Almost the same weight gain during growth process could have been emerged regardless of the difference in reliability of GEBV, because expected responses to selection for body weight in cattle are increase in weight at age of selection but also substantial increases in weight at all other ages [4]. Further research would be necessary to clarify the effect of inaccurate genetic parameter on expected genetic gain. More sophisticated procedures would be necessary to estimate genetic parameters for GEBV [31].

A selection index that was based on RR coefficients and imposed no restriction on selection intensity was developed [8]. In our point gain index, the procedure of [8] can be used to transform equation (1)***S***Δ***α****_L_* = Δ***G****_s_*, to ***S′S***Δ***α****_L_* = ***S′***Δ***G****_s_*, and Δ***α****_L_* = (**S′S**)**^−1^S′**Δ***G****_s_*. The difference in RR coefficients (Δ***α****_L_* = (**S′S**)**^−1^S′**Δ***G****_s_*) should be described to satisfy the desired gains. However, the difference in RR coefficients fails to achieve the desired gains (Δ***G****_s_*), because ***S***Δ***α****_L_* = ***S***(***S*′*S***)**^−1^*S*′**Δ***G****_s_* ≠ Δ***G****_s_*. The approach of [8] results in an index with minimum selection intensity only when the number of desired gains is equal to the number of RR coefficients fitted. In contrast, Δ***α****_L_* satisfies the equation of ***S*′*S***Δ***α****_L_* = ***S*′**Δ***G****_s_* for any number of desired gains and any order of fitted RR coefficients. Therefore, RR coefficients obtained by adding Δ***α****_L_* = (**S′S**)**^−1^S′**Δ***G****_s_* to the RR coefficients before selection is an option for obtaining new growth curve coefficients. However, the new growth curve needs to be checked to confirm that it at least nearly meets the breeder’s intention. This verification is necessary because this approach will not yield the desired gains unless the number of desired gains is equal to the order of the fitted RR coefficients.

A restricted selection index can achieve the desired gains in body weight for meat animals [32,33]. However, many possible growth curves could achieve the desired gains, but the effects on body weight throughout the entire fattening process will not be apparent unless all genetic correlations are known. Therefore, we applied a RR approach to the growth curve. As a result, our index approach offers a unique solution for achieving the desired gains with minimum selection intensity, revealing the effects on all body weights throughout the fattening process. The purpose of this study is not to apply RR curve to fattening process [10–13], but this study aims to develop selection index to achieve desired weight gains at targeted weeks of age during growth process minimizing inbreeding. Non-linear growth curve (Gompertz curve) have been applied to estimate breeding value [34,35], however, selection procedure for the desired weight gains at targeted weeks of age would not be given yet. The index was a general approach that is applicable to all species and accommodates weight increases at any age of animal or at any stage of the fattening process. The developed selection index procedure of the point gain index or the stage gain index would be extended easily to longitudinal data such as growth curve in plant and egg production curve.

## CONCLUSION

The point gain index we developed can achieve the desired weight gain at any given postnatal week of the growing process and is an easy-to-use and practical option for improving the growth curve. The index was a general approach that is applicable to all species and accommodates weight increases at any age of animal or at any stage of the fattening process. We presented a numerical example to illustrate our approach for reducing birth weight and reaching the desired finishing weight earlier than with other methods.

## Figures and Tables

**Table 1:** Genetic (co)variances of Legendre coefficients (kg)

Order	0	1	2	3	4	CV^1)^
0	4,502.4	1,367.7	−1,270.1	−25.2	227.1	0.112
1	-	1,062.8	67.7	−154.6	11.2	0.103
2	-	-	698.2	−88.6	−110.5	0.547
3	-	symmetric	-	38.1	10.0	0.270
4	-	-	-	-	18.3	0.363

1) Coefficient of variation = square root of genetic variance of Legendre coefficient/|Legendre coefficients before selection in Table 2|

**Table 2:** Birth weight (kg); body weight (kg) at 5, 81, 127, 128, and 130 weeks of age; and Legendre coefficients before selection and of the desired body weight curve

Item	Before selection	Desired curve
Birth weight	27.4	24.9
Body weight at (wk)		
5	40.2	38.8
81	570.7	589
127	713.5	718.4
128	715.8	720.6
130	720.4	725.8
Legendre coefficients		
α0^1)^	601.4	617.6
α1	317.9	320.1
α2	−48.3	−57.4
α3	−22.9	−22.3
α4	11.8	13.8

1) αi indicates order i.

**Table 3:** Age (weeks) and desired gain for 4 combinations of 3-stage gain and 4-point gain indices

Combination	Age (wk)	Desired gain (kg)	

		Stage gain index	Point gain index
1	0	-	−2.5
	40	231.5	15
	120	1,214.1	6.5
	130	44.5	5.4
2	0	-	−2.5
	26	62.4	8.5
	106	1,286.7	9.3
	130	141	5.4
3	0	-	−2.5
	26	62.4	8.5
	120	1,383.1	6.5
	130	44.5	5.4
4	0	-	−2.5
	42	262.6	15.7
	86	829.5	16.6
	130	398.1	5.4

**Table 4:** Selection intensity and achieved body weights due to index selection with desired increases (kg) for 4 points or 3 stages during the fattening process

Combination No^1)^	1			2		
	
	Stage gain index	Point gain index	Eigen vector index^2)^	Stage gain index	Point gain index	Eigen vector index
Selection intensity	0.412	0.21	0.21	0.43	0.358	0.358
Birth weight	24.3	24.9	24.9	23.5	24.9	24.9
Body weight at (wk)						
5	39.3	38.2	38.2	38.8	39.9	39.9
81	588.4	576.6	576.6	588.6	585.3	585.3
127	717	719.3	719.3	716.4	719.1	719.1
128	719	721.4	721.4	718.4	721.3	721.3
130	723.2	725.8	725.8	722.6	725.8	725.8

**Combination No**	**3**			**4**		
	
	**Stage gain index**	**Point gain index**	**Eigen vector index**	**Stage gain index**	**Point gain index**	**Eigen vector index**

Selection intensity	0.416	0.356	0.356	0.447	0.399	0.399
Birth weight	23.8	24.9	24.9	23.2	24.9	24.9
Body weight at (wk)						
5	39	39.9	39.9	38.5	40.1	40.1
81	588.5	585.6	585.6	588.8	588.3	588.3
127	717	719.1	719.1	715.8	719.3	719.3
128	719	721.3	721.3	717.8	721.4	721.4
130	723.2	725.8	725.8	722	725.8	725.8

1) Combination No corresponds to that of Table 3.

2) Number of index traits of eigenvector index is 5.

**Table 5:** Comparison of the number of eigenvectors in eigenvector index selection

Item	Eigenvector index				Point gain index
No of eigenvectors	5	4	3	2	-
Selection intensity	0.138	0.138	0.149	0.393	0.138
Difference between intended and achieved weight gain (kg)	0	0	0	0	0
Birth weight (kg)	24.9	24.9	24.9	24.9	24.9
Body weight (kg) at (wk)					
5	38	38	37.9	36	38
81	574.1	574	574.4	560	574.1
127	718.8	718.8	718.9	718.4	718.8
128	721.1	721.1	721.2	720.9	721.1
130	725.8	725.8	725.8	725.8	725.8

**Table 6:** Selection intensity and Legendre coefficients of stage gain, point gain, and eigenvector indices with different desired increases or restriction values

Index	Stage gain index		Point gain index		Eigenvector index (2 index traits)	
		
	1-stage		4-point		2-point	
		
	(0 to 130 wk)		(0, 42, 86, and 130 wk)		(0 and 130 wk)	
Restriction type	Actual weight	Scaled weight	Actual weight	Scaled weight	Actual weight	Scaled weight
Restriction values	1,490	1,000	−2.5, 15.7, 16.6, 5.4	−1, 6.28, 6.64, 2.16	−2.5, 5.4	−1, 2.16
Selection intensity	0.2895	0.1943	0.3988	0.1605	0.3929	0.1577
ΔL0^1)^/sd^2)^	0.24223	0.16257	0.25358	0.10135	−0.16690	−0.06658
ΔL1/sd	0.10498	0.07046	0.06072	0.02357	0.15107	0.06106
ΔL2/sd	−0.12171	−0.08168	−0.30193	−0.12026	0.26838	0.10766
ΔL3/sd	−0.00995	−0.00668	0.13205	0.05704	−0.18017	−0.07252
ΔL4/sd	0.13413	0.09002	0.22367	0.08973	−0.20290	−0.08131
b0^3)^	0.00517	0.00347	0.00284	0.00115	−0.00295	−0.00117
b1	−6.9 E–05	−4.62 E–05	0.00306	0.00133	0.01331	0.00535
b2	4.41 E–07	2.96 E–07	−0.01212	−0.00465		
b3	−0.00011	−7.06 E–05	0.02289	0.01073		
b4	3.01 E–06	2.021 E–06	−0.01118	−0.00435		

1) ΔL, Difference in Legendre coefficients (after selection – before selection): 0, constant; 1, linear; 2, quadratic; 3, cubic; 4, quartic.

2) sd, standard deviation.

3) b, index weight: 0, constant; 1, linear; 2, quadratic; 3, cubic; 4, quartic.

**Table 7:** Index coefficients, difference in Legendre coefficients (after selection – before selection, Δ*α_L_*), and selection intensity in reliability of genomically enhanced breeding value (0.7, 0.6, and 0.5)

Item	Index coefficients			Δ*α_L_*		
	
Reliability	0.7	0.6	0.5	0.7	0.6	0.5
Constant	0.00284	0.00390	0.00524	17.015	17.102	17.197
1st order	0.00306	0.00308	0.00343	1.980	1.990	2.002
2nd order	−0.01212	−0.01380	−0.01659	−7.978	−7.861	−7.734
3rd order	0.02289	0.02709	0.03331	0.815	0.808	0.801
4th order	−0.01118	−0.01233	−0.01449	0.958	0.842	0.716
Selection intensity	0.399	0.439	0.491	-	-	-

## Data Availability

All data generated or analyzed during this study are included in the published article.

## References

[1] Gregory KE (1965). Symposium on performance testing in beef cattle: Evaluating postweaning performance in beef cattle. J Anim Sci.

[2] Cartwright TC (1970). Selection criteria for beef cattle for the future. J Anim Sci.

[3] Dickerson GE, Künzi N, Cundiff LV, Koch RM, Arthaud VH, Gregory KE (1974). Selection criteria for efficient beef production. J Anim Sci.

[4] Fitzhugh HA (1974). Analysis of growth curves and strategies for altering their shape. J Anim Sci.

[5] Foulley JL (1976). Some considerations on selection criteria and optimization for terminal sire breeds. Genet Sel Evol.

[6] Schaeffer LR, Dekkers JCM Random regressions in animal models for test-day production in dairy cattle.

[7] Jamrozik J, Schaeffer LR, Dekkers JCM (1997). Genetic evaluation of dairy cattle using test day yields and random regression model. J Dairy Sci.

[8] Togashi K, Lin CY (2003). Modifying the lactation curve to improve lactation milk and persistency. J Dairy Sci.

[9] Togashi K, Lin CY (2004). Development of an optimal index to improve lactation yield and persistency with the least selection intensity. J Dairy Sci.

[10] Boligon AA, Mercadante MEZ, Forni S, Lôbo RB, Albuquerque LG (2010). Covariance functions for body weight from birth to maturity in Nellore cows. J Anim Sci.

[11] Meyer K (2004). Scope for a random regression model in genetic evaluation of beef cattle for growth. Livest Prod Sci.

[12] Meyer K (2005). Random regression analyses using B-splines to model growth of Australian Angus cattle. Genet Sel Evol.

[13] Mota LFM, Martins PGMA, Littiere TO, Abreu LRA, Silva MA, Bonafé CM (2018). Genetic evaluation and selection response for growth in meat-type quail through random regression models using B-spline functions and Legendre polynomials. Animal.

[14] Přibyl J, Krejčová1 H, Přibylová J, Misztal I, Bohmanová J, Štípková M (2007). Trajectory of body weight of performance tested dual-purpose bulls. Czech J Anim Sci.

[15] Togashi K, Adachi K, Kurogi K (2022). Predicting the rate of inbreeding in populations undergoing four-path selection on genomically enhanced breeding values. Anim Biosci.

[16] Togashi K, Lin CY (2006). Selection for milk production and persistency using eigenvectors of the random regression coefficient matrix. J Dairy Sci.

[17] Legarra A, Aguilar I, Misztal I (2009). A relationship matrix including full pedigree and genomic information. J Dairy Sci.

[18] Aguilar I, Misztal I, Johnson DL, Legarra A, Tsuruta S, Lawlor TJ (2010). Hot topic: a unified approach to utilize phenotypic, full pedigree, and genomic information for genetic evaluation of Holstein final score. J Dairy Sci.

[19] Christensen OF (2012). Compatibility of pedigree-based and marker-based relationship matrices for single-step genetic evaluation. Genet Sel Evol.

[20] Henderson CR (1984). Applications of linear models in animal breeding.

[21] Dekkers JCM (1992). Asymptotic response to selection on best linear unbiased predictors of breeding values. Anim Prod.

[22] Togashi K, Kurogi K, Adachi K (2020). Asymptotic response to four-path selection due to index and single trait selection according to genomically enhanced breeding values. Livest Sci.

[23] Searle SR (1966). Matrix Algebra for the Biological Science.

[24] Takeda M, Uemoto Y, Inoue K (2018). Evaluation of feed efficiency traits for genetic improvement in Japanese black cattle. J Anim Sci.

[25] Onogi A, Ogino A, Sato A, Kurogi K, Yasumori T, Togashi K (2019). Development of a structural growth curve model that considers the causal effect of initial phenotypes. Genet Sel Evol.

[26] Teixeira BB, Mota RR, Lôbo RB (2018). Genetic evaluation of growth traits in nellore cattle through multi-trait and random regression models. Czech J Anim Sci.

[27] Ferreira JL, Lopes FB, Pereira LS (2015). Estimation of (co)variances for growth traits in Nellore cattle raised in the Humid Tropics of Brazil by random regression. Ciênc Agrár.

[28] National Livestock Breeding Center (c2022). The trend of genetic ability in Japanese Black cattle.

[29] Harris DL (1964). Expected and predicted progress from index selection involving estimates of population parameters. Biometrics.

[30] Heidhues T (1961). Relative accuracy of selection indices based on estimated genotypic and phenotypic parameters. Biometrics.

[31] Misztal I (2023). Estimation of heritabilities and genetic correlations by time slices using predictivity in large genomic models. bioRxiv.

[32] James JW (1968). Index selection with restrictions. Biometrics.

[33] Kempthorne O, Nordskog AW (1959). Restricted selection indices. Biometrics.

[34] Ma J, Chen J, Gan M (2022). Gut microbiota composition and diversity in different commercial swine breeds in early and finishing growth stages. Animals (Basel).

[35] Koivula M, Sevón-Aimonen ML, Strandén I (2008). Genetic (co)variances and breeding value estimation of Gompertz growth curve parameters in Finnish Yorkshire boars, gilts and barrows. J Anim Breed Genet.

