# Supplementary material for: Development of an index that decreases birth weight, promotes postnatal growth and yet minimizes selection intensity in beef cattle
Source: Anim Biosci. 2024 Jan 20;37(5):839–51. doi: 10.5713/ab.23.0343 (PMC11065704; doi:10.5713/ab.23.0343)
Supplement: Supplementary file 1 [file ab-23-0343-Supplementary-1.pdf]

Supplementary file

Appendix: Estimation of random regression parameters from parameters from Gompertz growth curve

The weekly genetic covariance matrix (***G***) from birth through 130 weeks of age was obtained by generating random numbers based on the genetic parameters of the Gompertz growth curve.

The weekly genetic covariance matrix (***G***) was transformed as follows;

$$\boldsymbol{G} = \boldsymbol{\varphi H \varphi'}$$

,where ***H*** =the genetic covariance matrix of Legendre polynomial coefficients,

$$\boldsymbol{\varphi} = \begin{bmatrix} \varphi_0(t_0) & \varphi_1(t_0) & \varphi_2(t_0) & .. & \varphi_k(t_0) \\ \varphi_0(t_1) & \varphi_1(t_1) & \varphi_2(t_1) & .. & \varphi_k(t_1) \\ .. & .. & .. & .. & .. \\ \varphi_0(t_{130}) & \varphi_1(t_{130}) & \varphi_2(t_{130}) & .. & \varphi_k(t_{130}) \end{bmatrix},$$

$\boldsymbol{\varphi}$ is a $131 \times (k+1)$ matrix, t_i is the age standardized for the ith specific time in fattening process and $\varphi_{\boldsymbol{j}}(t_i)$ is the jth order of Legendre polynomial (*j* = *0,..,k*) evaluated at age t_i standardized,

$\boldsymbol{\varphi}$ is defined by Legendre polynomial functions and does not depend on the values in the matrix ***G***. Therefore, it is possible to estimate ***H*** as follows;

$$\boldsymbol{H} = \boldsymbol{\varphi}^{-1}\boldsymbol{G}(\boldsymbol{\varphi}^{-1})'.$$
